# Hafnium-Based Metal–Organic Framework Nanoparticles as a Radiosensitizer to Improve Radiotherapy Efficacy in Esophageal Cancer

**DOI:** 10.1021/acsomega.2c00223

**Published:** 2022-03-30

**Authors:** Wei Zhou, Zhulong Liu, Nana Wang, Xue Chen, Xiaozheng Sun, Yufeng Cheng

**Affiliations:** Department of Radiation Oncology, Cheeloo College of Medicine, Qilu Hospital, Shandong University, Jinan 250100, China

## Abstract

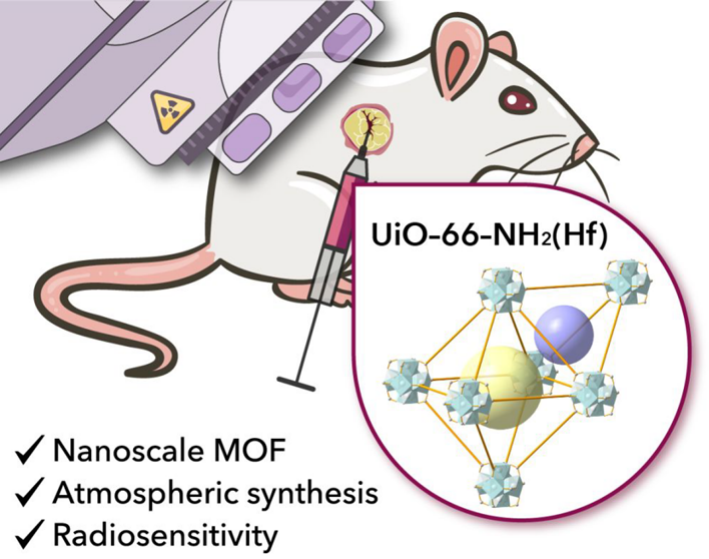

Radiotherapy is one of the most widely used clinical treatments for tumors, but it faces limitations, such as poor X-ray retention at the tumor site. The use of radiosensitizers containing high *Z* elements is an effective way to enhance X-ray absorption. Here, we demonstrate a simple one-step method for the synthesis of UiO-66-NH_2_(Hf) metal–organic framework nanoparticles for use as radiosensitizers in radiotherapy. The UiO-66-NH_2_(Hf) nanoparticles had a diameter of less than 100 nm and were stable in the physiological environment. UiO-66-NH_2_(Hf) induced apoptosis by enhancing X-ray absorption, as confirmed by *in vitro* and *in vivo* experiments. These characteristics make UiO-66-NH_2_(Hf) a promising radiosensitizer for esophageal cancer radiotherapy.

## 1 Introduction

Radiotherapy, as one of the most widely used tumor therapies in the clinic, typically employs high-energy ionizing radiation (e.g., X-rays and γ-rays) to destroy biomolecules within tumor tissues, including DNA and proteins, to induce tumor ablation.^1^ Radiotherapy has the advantages of being noninvasive, exhibiting deep tissue penetration, and having precise and controllable localization compared to chemotherapy.^2^ However, radiotherapy also suffers from a range of problems. First, the absorption of radiation in tumors remains low, which greatly limits the dose of radiation that can be administered.^3^ Second, the tumor microenvironment, which is mainly characterized by hypoxia, exacerbates radiotherapy resistance,^4^ and widespread resistance mechanisms (e.g., DNA repair and epithelial–mesenchymal transition) in tumors further erode the therapeutic effect.^5^ Therefore, investigations into effective ways to improve the efficacy of radiotherapy and minimize adverse effects on tumor-adjacent normal tissues are urgently needed.

Currently, there are two main strategies for radiotherapy sensitization. The first strategy is to induce radiosensitization with chemotherapeutic drugs. For example, dichloroacetate enhances the sensitivity of A549 lung cancer cells to X-rays by attenuating aerobic glycolysis.^6^ Nitroimidazoles improve the sensitivity of hypoxic tumors to radiation by inhibiting DNA damage repair.^7^ However, this strategy is essentially chemotherapy, and systemic toxicity is difficult to avoid. The second strategy is to increase the radiation energy deposition in tumor tissue through high *Z* elements (e.g., Au, Bi, and I).^8^ For example, iopromide and iohexol have a high X-ray absorption capacity due to their high iodine content, but they are more suitable for computed tomography (CT) imaging of blood vessels than radiotherapy sensitization due to difficulties related to penetrating the vascular wall to enter tumor tissues.^9^ The ability to enhance radiotherapy with nanomaterials containing high *Z* elements, including gold nanoparticles,^10^ BiOI,^11^ CeO_2_,^12^ and rare earth ions,^13^ has been demonstrated previously. However, the main drawback of these inorganic nanomaterials is that they are difficult to degrade *in vivo* and may cause long-term toxicity issues.^14^

Metal–organic frameworks (MOFs) are crystalline porous materials formed by the self-assembly of organic ligands and metal ions driven by coordination bonds.^15^ MOFs have good biocompatibility, which has been shown in a variety of biomedical applications.^16^ Recently, Lin et al. reported the application of various Hf-based MOFs as X-ray-absorbing reagents for colon cancer therapy in mice.^17^ Hf-based MOFs can be easily combined with other therapies, such as photodynamic therapy^18^ and immunotherapy,^19^ to further enhance cancer treatment. However, there are several limitations related to the preparation of these MOF-based materials; they include the use of eco-unfriendly organic solvents, harsh solvothermal reaction conditions, complicated multistep preparation processes, and issues with mass preparation, which are potential obstacles to clinical application.^20^

**Figure 1:**
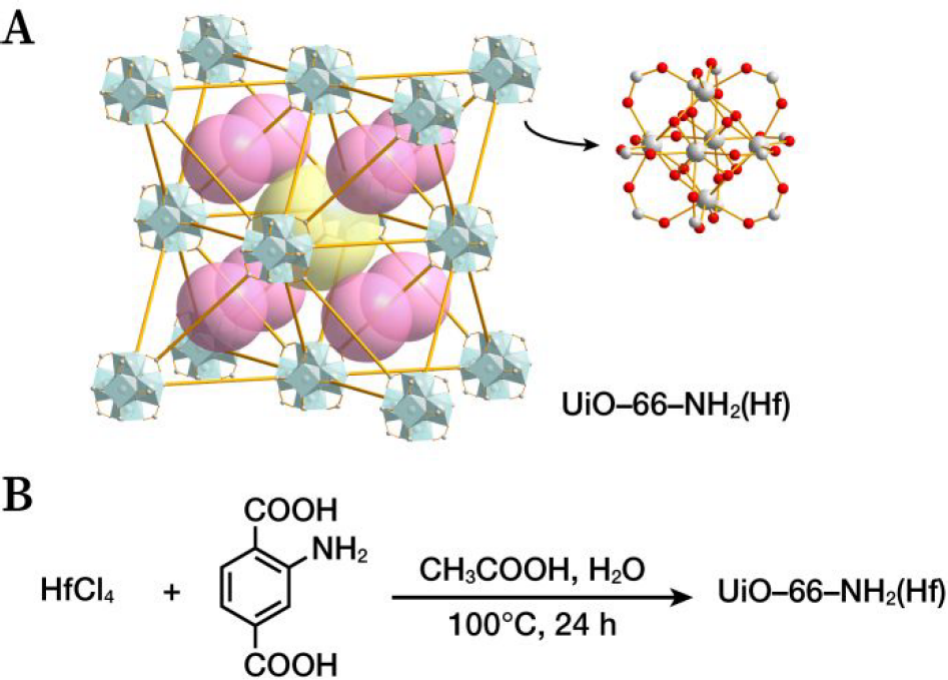
(A) Structure of UiO-66-NH_2_(Hf). The white and red balls represent Hf atoms and oxygen atoms, respectively. The yellow and pink balls represent the octahedral pores and tetrahedral pores of UiO-66-NH_2_(Hf), respectively. (B) Synthesis of UiO-66-NH_2_(Hf) in water.

Herein, we synthesized the Hf-based MOF UiO-66-NH_2_(Hf) in an aqueous solution under atmospheric pressure conditions and applied it as a radiosensitizer in the treatment of esophageal cancer (Figure 1). Without further modifications, the obtained UiO-66-NH_2_(Hf) MOF elicited good anticancer effects *in vitro* and *in vivo* by increasing X-ray absorption in malignant tissues. Our study emphasizes that basic materials can meet the needs of biomedical applications without complicated synthesis steps, bridging the gap between laboratory materials science and clinical oncology research.

## 2 Experimental Section

### 2.1 Synthesis of UiO-66-NH_2_(Hf)

Amounts of 3.36 g of HfCl_4_ (Macklin, Cat# H888681) and 1.81 g of 2-aminoterephthalic acid (TCI, Cat# A1291) were added to 80 mL of acetic acid and 120 mL of water. The solution was heated to 100 °C and stirred for 24 h. After the reaction was completed, the milky white suspension was cooled to room temperature and centrifuged at 13 300 rpm for 10 min to collect the product. Then, the product was washed three times with water and three times with ethanol. Finally, the product was dried at 70 °C for 12 h. The product was a white powder, and the yield was 80%.

### 2.2 Cell Viability Assays

KYSE 150 cells were cultured at a density of 5000 cells/well in 96-well plates for 24 h, treated with UiO-66-NH_2_(Hf) (100 μL, 0–200 μg/mL) for 4 h, and exposed to X-ray irradiation (0–8 Gy). After 24 h, the culture medium (90 μL) and a CCK-8 solution (10 μL) were added to each well, and the plate was incubated in a CO_2_ incubator for approximately 2 h. The absorbance at 450 nm was measured using a Molecular Devices SpectraMax i3x multimode microplate detection system. Cells treated with neither UiO-66-NH_2_(Hf) nor X-ray irradiation were used as controls.

### 2.3 Clonogenic Assay

KYSE 150 cells were cultured at a density of 2000 cells/well in 6-well plates for 24 h, treated with UiO-66-NH_2_(Hf) (1.0 mL, 0 or 50 μg/mL) for 4 h, and exposed to X-ray irradiation (0 or 6 Gy). After approximately 2 weeks, the cells were fixed with 4% paraformaldehyde and stained with 0.1% crystal violet. The plates were washed, air-dried, and photographed with a digital camera. Cells treated with neither UiO-66-NH_2_(Hf) nor X-ray irradiation were used as controls.

### 2.4 γH2AX Immunofluorescence Staining

KYSE 150 cells were cultured in glass-bottom dishes for 24 h, treated with UiO-66-NH_2_(Hf) (1.0 mL, 0 or 50 μg/mL) for 4 h, and exposed to X-ray irradiation (0 or 6 Gy). After 3 h, the cells were fixed in 4% paraformaldehyde for 1 h, permeabilized with Triton X-100 (0.5 vol %) for 5 min, incubated in normal goat serum (5 vol %) for 1 h, and then incubated with an anti-γH2AX primary antibody (1:400, Cell Signaling Technology, Cat# 9718) at 4 °C overnight. The cells were washed three times with phosphate-buffered saline (PBS) and incubated with a DyLight 594-labeled secondary antibody (1:500, Invitrogen, Cat# 35560) for 1 h at room temperature. Finally, the cell nuclei were counterstained with Hoechst 33342 (20 μM). Laser scanning confocal fluorescence images were acquired. Cells treated with neither UiO-66-NH_2_(Hf) nor X-ray irradiation were used as controls.

### 2.5 Intracellular ROS Measurements

KYSE 150 cells were treated with UiO-66-NH_2_(Hf) (0 or 50 μg/mL) for 4 h and exposed to X-ray irradiation (0 or 6 Gy). After 12 h, the cells were stained with 2,7-dichlorodihydrofluorescein diacetate (DCFH-DA, 20 μM) for 30 min and collected using a trypsin-EDTA solution. The green fluorescence of DCFH-DA was detected on a BD FACSCalibur flow cytometer using the FL1 channel. Cells treated with neither UiO-66-NH_2_(Hf) nor X-ray irradiation were used as controls.

### 2.6 Apoptosis Analysis

KYSE 150 cells were treated with UiO-66-NH_2_(Hf) (0 or 50 μg/mL) for 4 h and exposed to X-ray irradiation (0 or 6 Gy). After 12 h, the cells were stained with annexin V-allophycocyanin (APC) and 7-aminoactinomycin D (7-AAD) according to the manufacturer’s guidelines and collected using an EDTA-free trypsin solution. Cell apoptosis was detected on a BD FACSCalibur flow cytometer. Cells treated with neither UiO-66-NH_2_(Hf) nor X-ray irradiation were used as controls.

### 2.7 Western Blotting

KYSE 150 cells were treated with UiO-66-NH_2_(Hf) (0 or 50 μg/mL) for 4 h and exposed to X-ray irradiation (0 or 6 Gy). After 24 h, total protein was extracted using RIPA lysis and extraction buffer (Thermo Scientific, Cat# 89900) and quantified with a BCA protein assay kit (Thermo Scientific, Cat# 23227). An amount of 30 micrograms of protein per sample was loaded and subjected to electrophoresis to separate the target protein. After transferring the separated proteins to a polyvinylidene difluoride membrane and blocking for 1 h using 5% nonfat powdered milk, the membrane was incubated with primary antibodies against Bax (Abcam, Cat# ab32503) and Bcl-2 (Abcam, Cat# ab182858) at 4 °C overnight. Then, the protein of interest was identified with an HRP-conjugated antirabbit secondary antibody (Proteintech, Cat# SA00001-2) and a chemiluminescence detection kit (Vazyme, Cat# E412-01). Cells treated with neither UiO-66-NH_2_(Hf) nor X-ray irradiation were used as controls.

### 2.8 Animal Experiments

Female BALB/c nude mice (4 weeks old) were purchased from Charles River (Beijing, China). All animal procedures were reviewed and approved by the Animal Ethical Committee of Qilu Hospital of Shandong University. KYSE 150 cells (approximately 10^6^ cells) suspended in PBS (100 μL) were subcutaneously injected into the flanks of each BALB/c nude mouse to establish a KYSE 150 xenograft model. The length (*L*) and width (*W*) of each tumor were determined using digital calipers. The tumor volume (*V*) was calculated using the following formula: *V* = 1/2 × *L* × *W*^2^.

When the tumor size reached approximately 50 mm^3^, mice (*n* = 16) were randomly divided into 4 groups and injected intratumorally with UiO-66-NH_2_(Hf) (40 μL, 0 or 2.0 mg/mL). After 2 h, the mouse xenografts were exposed to X-ray irradiation (0 or 8 Gy). Then, the mice were housed for 8 days before euthanasia, and the tumors were collected.

For CT imaging, when the tumor size reached approximately 300 mm^3^, mice (*n* = 8) were randomly divided into 2 groups and injected intratumorally with UiO-66-NH_2_(Hf) (40 μL, 0 or 2.0 mg/mL). After 2 h, the mice were anesthetized, and CT imaging was performed on a SOMATOM Force CT (Siemens, Germany).

### 2.9 Statistics

Statistical analyses were performed with GraphPad Prism V.9.3 statistical software. Data are presented as the mean ± SD unless otherwise indicated. Data were compared using an unpaired Student’s *t* test and a one- or two-way analysis of variance (ANOVA) followed by Tukey’s post hoc test, as appropriate. Statistical significance was annotated as follows: *p < 0.05, **p < 0.01, ***p < 0.001, and ****p < 0.0001.

## 3 Results

### 3.1 Synthesis and Characterization

UiO-66-NH_2_(Hf), as an analogue of UiO-66(Zr),^21^ is a three-dimensional porous framework composed of hexanuclear octahedral hafnium oxoclusters linked by 2-aminoterephthalate (Figure 1A).^22^ UiO-66-NH_2_(Hf) is conventionally synthesized by the solvothermal method, in which the synthetic reaction is promoted by autogenous pressure generated by heating an organic solvent in a closed vessel.^23^ In contrast to the usual solvothermal method, atmospheric pressure conditions were used to produce UiO-66-NH_2_(Hf) in this study. Specifically, UiO-66-NH_2_(Hf), as a white powder, was obtained at a high yield of approximately 80% by reacting HfCl_4_ as the hafnium source, 2-aminoterephthalic acid as the organic ligand, and acetic acid as the regulator at 100 °C for 24 h in an aqueous solution (Figure 1B).

**Figure 2:**
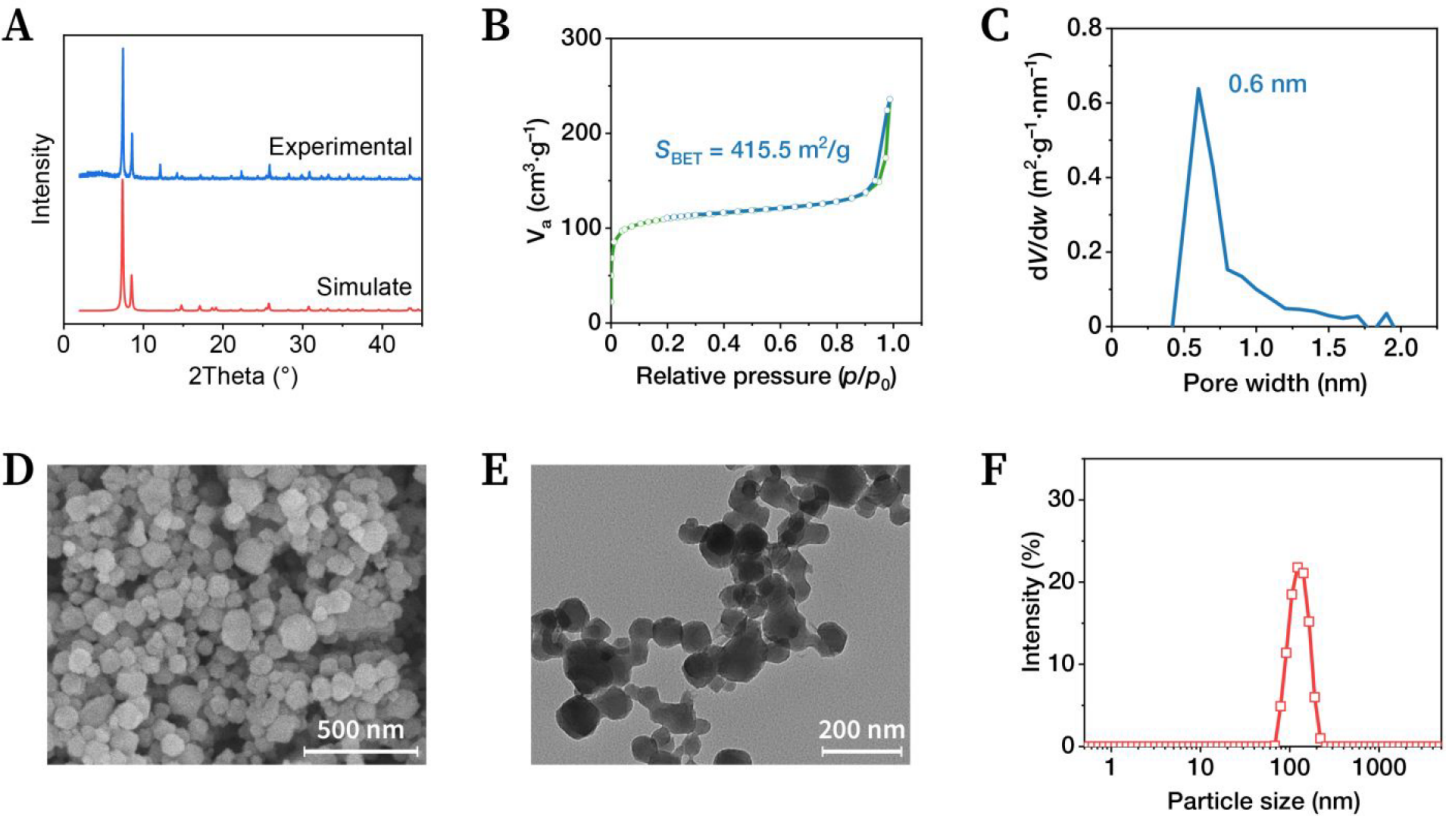
Material characterization. (A) Simulated and experimental powder X-ray diffraction (PXRD) patterns. (B) N_2_ adsorption and desorption isotherms at 77 K. (C) Pore width distribution plot. (D) SEM image of UiO-66-NH_2_(Hf). Magnification: 50 000×. (E) TEM image of UiO-66-NH_2_(Hf). Magnification: 30 000×. (F) Particle size distribution plot based on dynamic light-scattering measurements.

The powder X-ray diffraction (PXRD) pattern exhibited two main diffraction peaks attributed to the (111) and (002) reflections, located at 7.44° and 8.59°, respectively, which were consistent with the simulated results and reported in the literature (Figure 2A).^24^ An additional broad peak was observed in the region centered at 4.7°, which might be related to the absence of hafnium oxyclusters.^25^ The crystallinity of UiO-66-NH_2_(Hf) was further confirmed by N_2_ adsorption and desorption experiments performed at 77 K (Figure 2B). The N_2_ adsorption isotherm had a clear type I characteristic, and the total pore volume was 0.365 cm^3^/g at *p*/*p*_0_ = 0.987, which both indicated microporous property. The Brunauer–Emmett–Teller (BET) specific surface area was determined to be 415.5 m^2^/g according to the BET equation, implying the existence of a permanent pore space. Micropore analysis identified a pore size of 0.6 nm, consistent with the theoretical value (Figure 2C).

An appropriate particle size is critical for biomedical applications. Based on scanning electron microscopy (SEM) images, the UiO-66-NH_2_(Hf) generated in this study had a more rounded microscopic morphology (Figure 2D), which approximated a spherical shape, unlike the octahedral-shaped particles obtained by the solvothermal reaction.^26^ Transmission electron microscopy (TEM) imaging obtained the same results as SEM imaging (Figure 2E). After random inspection of 200 particles in TEM images, the average particle size was calculated to be 95 ± 18 nm. Dynamic light scattering (DLS) indicated a hydrodynamic size of 127.8 nm, which was attributed to strong hydrophilicity (Figure 2F). According to time-dependent PXRD and DLS measurements, UiO-66-NH_2_(Hf) at a concentration of 10 mg/mL was stable for at least 24 h without significant aggregation in PBS and Dulbecco’s modified Eagle’s medium (DMEM), satisfying the requirements for biomedical applications (Figure S1).

### 3.2 Enhanced Radiotherapy *in Vitro*

**Figure 3:**
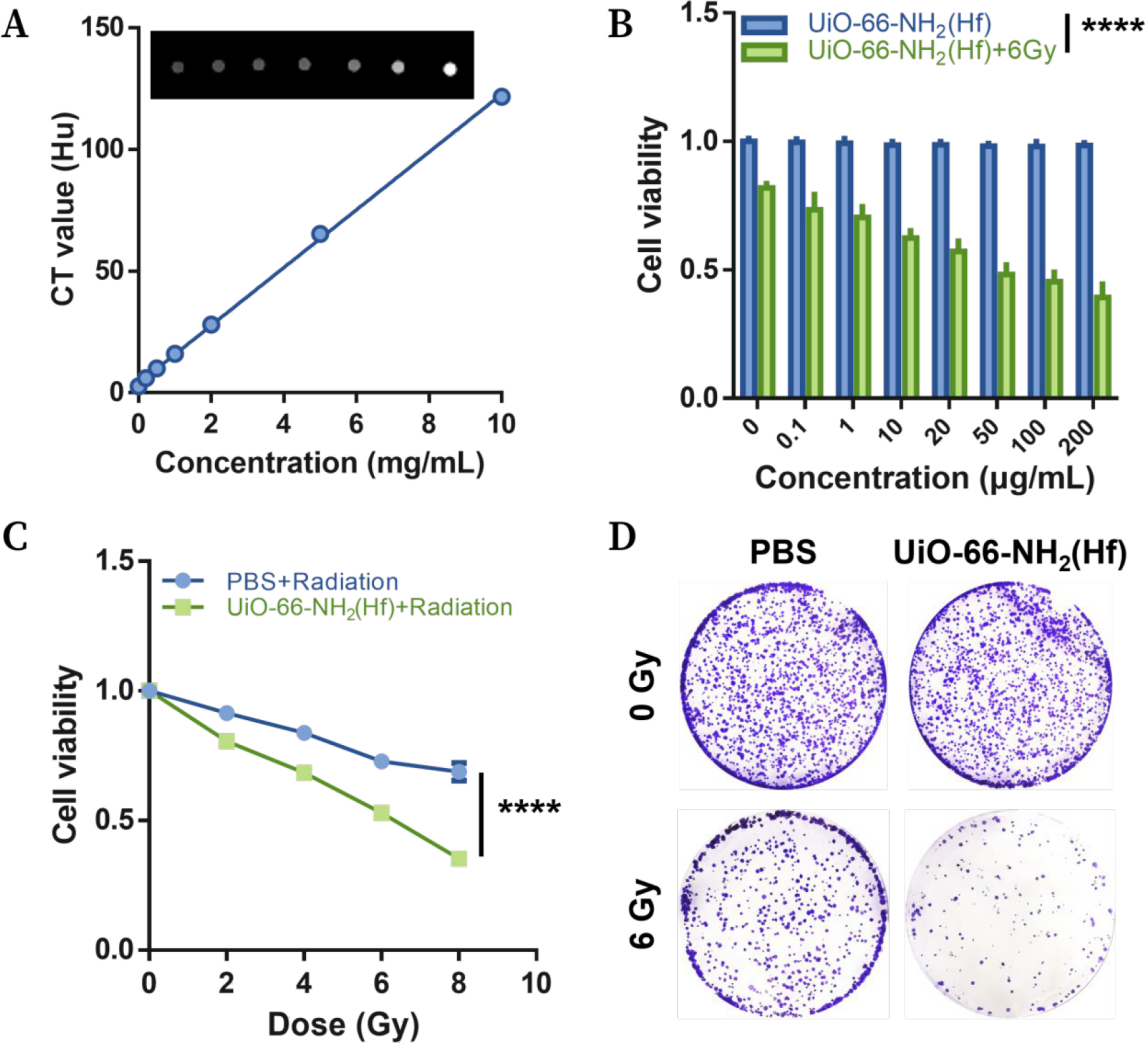
UiO-66-NH_2_(Hf) improved radiotherapy efficacy against KYSE 150 cells by increasing X-ray absorption. (A) The linear relationship between the CT value and the concentration of UiO-66-NH_2_(Hf) in a PBS dispersion. (B) Cell viabilities of KYSE 150 cells treated with UiO-66-NH_2_(Hf) (0–200 μg/mL) for 4 h and exposed to 0 or 6 Gy X-ray irradiation. (C) Cell viabilities of KYSE 150 cells treated with UiO-66-NH_2_(Hf) (0 or 50 μg/mL) for 4 h and exposed to 0–8 Gy X-ray irradiation. (D) Clonogenic assay performed with KYSE 150 cells treated with UiO-66-NH_2_(Hf) (0 or 50 μg/mL) for 4 h and exposed to 0 or 6 Gy X-ray irradiation. Data are presented as the mean ± SD; *n* = 3 (A) and *n* = 5 (B and C). Significance was determined by two-way ANOVA. ***p < 0.001, ****p < 0.0001.

In view of the high Hf content in UiO-66-NH_2_(Hf) (theoretical value of 47.0 wt % and measured value of 44.1 wt % based on inductively coupled plasma–optical emission spectrometry), we next investigated the X-ray attenuation property of UiO-66-NH_2_(Hf). CT scans of UiO-66-NH_2_(Hf) in PBS were performed, and a good linear relationship between the CT value and concentration was observed: *Y* = 11.88*C*/(mg/mL) + 3.924, *R*^2^ = 0.9990 (Figure 3A). The specific CT value was 4.9 × 10^3^ Hu/M (Hf equiv), which was comparable to that of the commercial CT contrast agent iodixanol (5.4 × 10^3^ Hu/M, I equiv),^27^ indicating the significant X-ray absorption ability of UiO-66-NH_2_(Hf).

Subsequently, the radiosensitizing effect of UiO-66-NH_2_(Hf) on the esophageal squamous cell line KYSE 150 was investigated. As shown in Figure 3B, a PBS dispersion of UiO-66-NH_2_(Hf) at a concentration of up to 200 μg/mL was used to treat KYSE 150 cells for 4 h, leading to a cell viability rate of 98.5 ± 0.9%, indicating that UiO-66-NH_2_(Hf) did not cause a discernible adverse effect on cell viability. However, cell viability was significantly reduced to 39.3 ± 0.5% when the treated cells were exposed to a 6 Gy X-ray irradiation, indicating that UiO-66-NH_2_(Hf) enhanced the killing effect of X-ray irradiation on the cells. This enhancement was concentration dependent, further implying that the increased X-ray absorption by the material was responsible for the decrease in cell viability. Not surprisingly, cell viability decreased with increasing X-ray doses, and this decrease was exacerbated by UiO-66-NH_2_(Hf), again suggesting a boosting effect of UiO-66-NH_2_(Hf) on radiotherapy efficacy (Figure 3C).

A clonogenic assay can be performed to evaluate cell death after ionizing radiation treatment; this assay evaluates the ability of a single cell to grow into a colony consisting of at least 50 cells.^28^ As shown in Figure 3D, UiO-66-NH_2_(Hf) treatment exhibited no adverse effect on cell clone formation compared to control treatment. Exposure of cells to 6 Gy X-ray irradiation resulted in diminished clone formation, and pretreatment of cells with UiO-66-NH_2_(Hf) before exposure resulted in the most obvious reduction in the number of clones. All of these findings were consistent with those obtained by CCK-8 analysis, indicating the enhancing effect of UiO-66-NH_2_(Hf) on the effects of radiotherapy on KYSE 150 cells.

Furthermore, a wound healing assay can simulate the tumor migration process *in vitro* and is an effective method for studying cell migration. As shown in Figure S2, as expected, wounds in untreated cell monolayers were almost completely healed at 48 h after artificial wounding, with or without UiO-66-NH_2_(Hf). After exposure to 6 Gy X-ray irradiation, scratches failed to close completely within 48 h, and cells migrated slowly after being pretreated with UiO-66-NH_2_(Hf). In brief, the scratch experiments showed that UiO-66-NH_2_(Hf) could improve radiotherapy efficacy by preventing cell migration.

**Figure 4:**
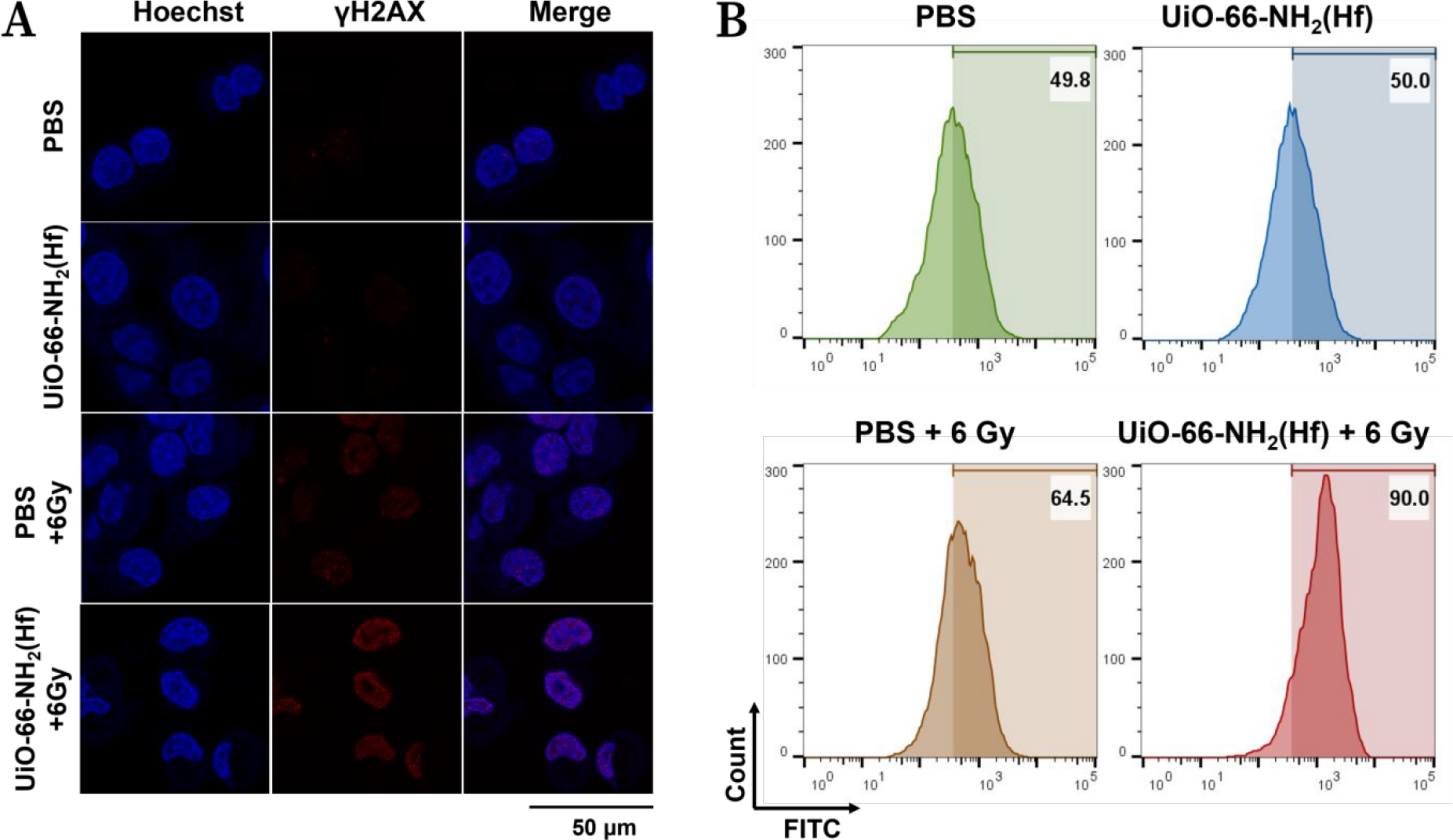
Upregulated DNA damage and intracellular ROS. (A) γH2AX immunofluorescence staining of KYSE 150 cells exposed to UiO-66-NH_2_(Hf) (0 or 50 μg/mL) for 4 h with or without 6 Gy X-ray irradiation. (B) Flow cytometric analysis of ROS generation in KYSE 150 cells incubated with UiO-66-NH_2_(Hf) (0 or 50 μg/mL) for 4 h with or without 6 Gy X-ray irradiation.

Phosphorylation of the Ser139 residue of H2A histone family member X (H2AX) to form γH2AX is an early event in DNA double-stranded breaks and is considered a highly sensitive and specific biomarker for monitoring X-ray-induced DNA damage.^29^ Immunofluorescence staining showed that KYSE 150 cells treated with UiO-66-NH_2_(Hf) and exposed to a 6 Gy dose of X-ray irradiation had a distinct fluorescence punctate in the nucleus, which was stronger than that in cells treated with only X-ray irradiation (Figure 4A). This enhancement resulted from additional DNA breaks caused by the elevated absorption of X-rays by UiO-66-NH_2_(Hf). In addition, DNA damage inevitably leads to oxidative stress as a downstream event, which manifested as the upregulation of intracellular reactive oxygen species (ROS) indicated by flow cytometry results for DCFH-DA staining (Figure 4B).

**Figure 5:**
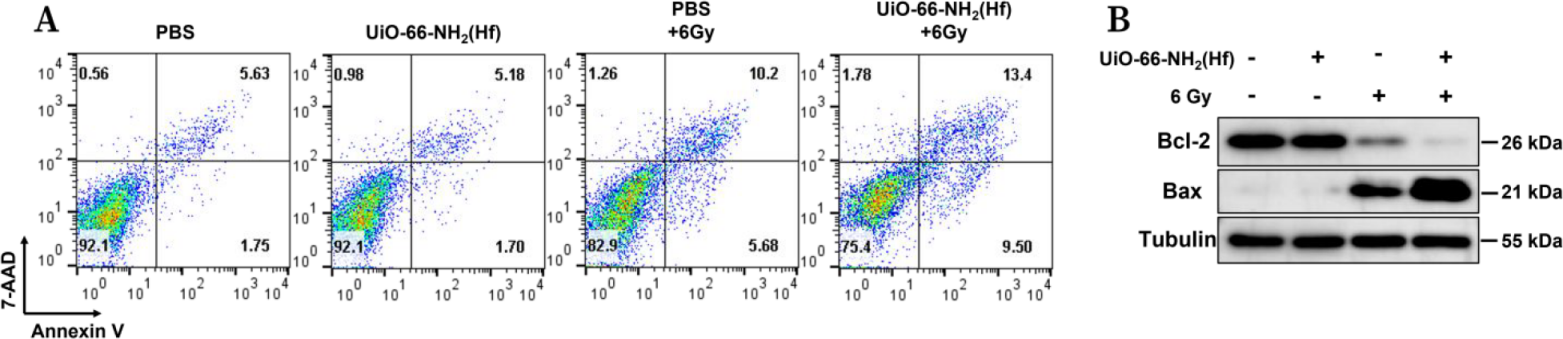
UiO-66-NH_2_(Hf)-promoted cell apoptosis. (A) Flow cytometric analysis of apoptosis in KYSE 150 cells incubated with UiO-66-NH_2_(Hf) (0 or 50 μg/mL) for 4 h with or without 6 Gy X-ray irradiation. (B) Detection of the expression of Bcl-2 and Bax in KYSE 150 cells pretreated with UiO-66-NH_2_(Hf) (0 or 50 μg/mL) for 4 h and exposed to 0 or 6 Gy X-ray irradiation. The uncropped Western blot images are shown in Figure S4.

Inspired by the above results, we investigated the cell death pathway induced by the combination of UiO-66-NH_2_(Hf) and X-ray irradiation. Under normal conditions, phosphatidylserine (PS) is located mainly on the inner side of the plasma membrane, but when apoptosis begins, PS is transferred from the inner side to the outer side. APC-labeled annexin V has a high affinity for PS and can mark cells undergoing apoptosis. 7-AAD is a membrane-impermeable fluorescent dye that can be used to label dead cells. As shown in Figures 5A and S3, annexin V-APC and 7-AAD double staining was used to measure early phase apoptotic cells induced by cotreatment of UiO-66-NH_2_(Hf) and X-ray irradiation using flow cytometry, and the results showed an elevated trend for apoptosis, consistent with the results for γH2AX and ROS described above. In addition, the protein levels of Bcl-2 and Bax in cells treated under different conditions were analyzed (Figure 5B). It has been shown that Bcl-2 inhibits apoptosis, while Bax is proapoptotic and acts by forming a dimer with Bcl-2 to prevent the inhibitory effect of Bcl-2 on apoptosis. Western blot analysis showed that UiO-66-NH_2_(Hf) pretreatment increased Bax expression while decreasing Bcl-2 expression in cells exposed to X-ray irradiation, indicating an elevated level of apoptosis with pretreatment compared to control treatment.

In conclusion, the *in vitro* results strongly suggested that UiO-66-NH_2_(Hf) promoted apoptosis by facilitating X-ray absorption, thus favoring radiotherapy efficacy.

### 3.3 Enhanced CT Imaging and Radiotherapy *in Vivo*

**Figure 6:**
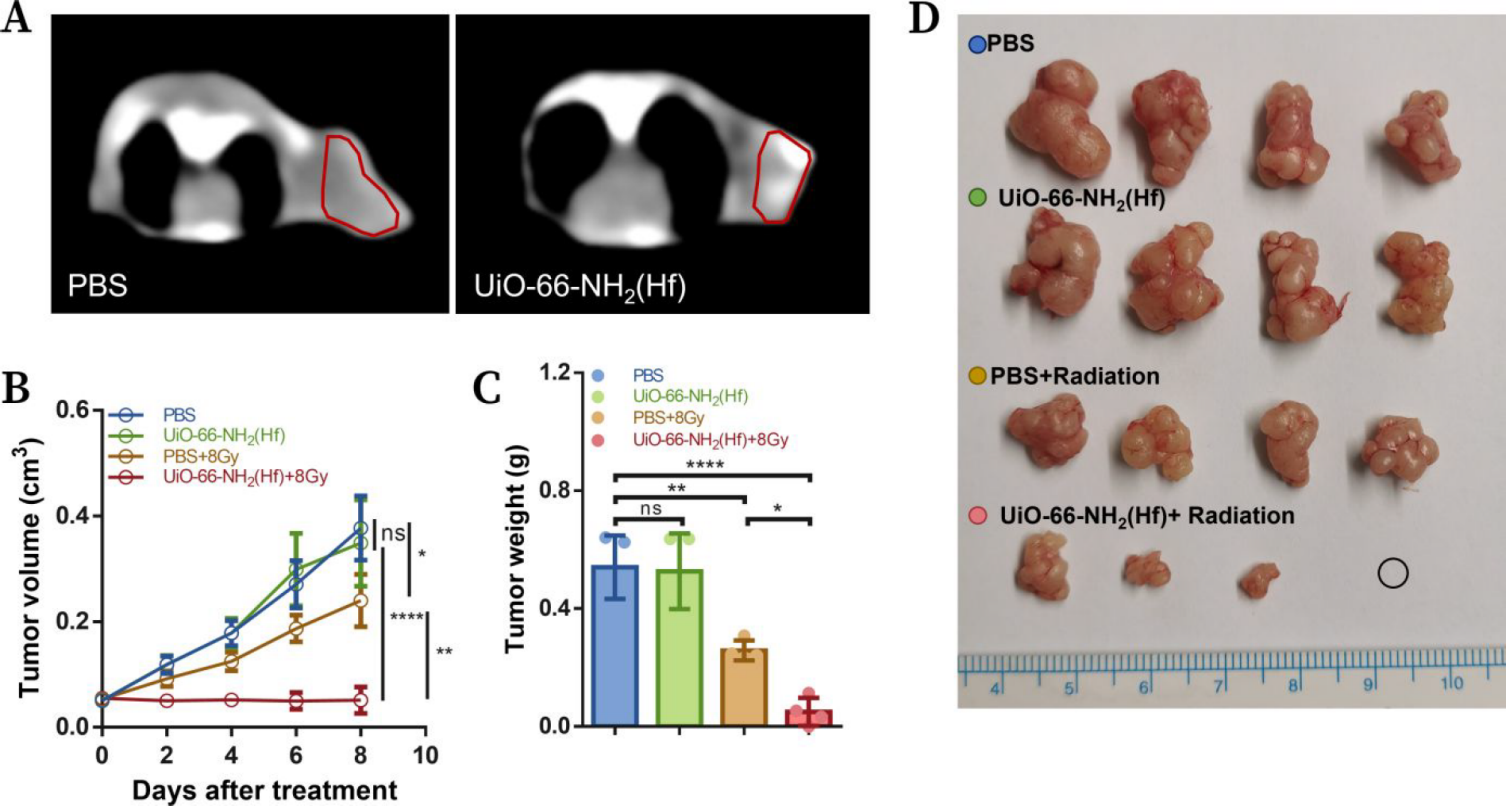
*In vivo* therapeutic effect. (A) CT images of KYSE 150 tumor-bearing nude mice after intratumoral injection of UiO-66-NH_2_(Hf) (2.0 mg/mL). (B) Tumor growth curves of xenografts after treatment with UiO-66-NH_2_(Hf) (0 or 2.0 mg/mL) and/or irradiation (0 or 8 Gy). (C) Excised tumor weight on day 8 after treatment. (D) Photographs of tumors excised on day 8 after treatment. Data are presented as the mean ± SD, *n* = 4. Significance was determined by two-way ANOVA (B) or one-way (C) ANOVA with Tukey’s multiple comparison test. *p < 0.05; **p < 0.01; ****p < 0.0001; ns, no significance (*p* > 0.05).

We further studied the effect of UiO-66-NH_2_(Hf) as a radiosensitizer in tumor treatment using a KYSE 150 xenograft model. Intratumoral injection of UiO-66-NH_2_(Hf) resulted in an increase in CT values at the tumor site from 96 Hu to 151 Hu in nude mice (Figure 6A), indicating effective X-ray deposition *in vivo*. When the tumor volume reached 50 mm^3^, mice were treated with UiO-66-NH_2_(Hf) administered intratumorally, or radiotherapy was administered locally to tumor sites and then housed for 8 days. The tumor growth curves showed that combined treatment with UiO-66-NH_2_(Hf) and a single fraction of X-ray irradiation almost stopped tumor growth, while X-ray irradiation alone resulted in only a limited delay in tumor growth. UiO-66-NH_2_(Hf) alone did not produce any effect on tumor growth (Figure 6B). These results were in good agreement with the earlier *in vitro* experiments. All the mice were sacrificed on the eighth day after treatment, and the weight and size of the tumors obtained were consistent with the observations during the breeding period (Figure 6C,D). In particular, the tumor of one mouse completely disappeared after treatment with UiO-66-NH_2_(Hf) and radiotherapy, suggesting the great potential of UiO-66-NH_2_(Hf) as a radiosensitizer. Finally, no significant weight loss was observed in any mice throughout the experiments (Figure S5), indicating the negligible systemic toxicity of UiO-66-NH_2_(Hf) in the short term.

## 4 Discussion

Esophageal cancer is the seventh most frequent cancer worldwide and the sixth leading cause of cancer-related death.^30^ Esophageal cancer patients have a poor prognosis even with comprehensive treatment regimens, as evidenced by the 5-year survival rate of 15–25% and the recurrence rate of over 40% after radical surgery.^31^ Surgery, radiotherapy, and chemotherapy are currently the most common and established forms of treatment for esophageal cancer, among which radiotherapy is not only a radical treatment choice but also an important adjunct for other treatments for esophageal cancer.^32^ However, radioresistance is an intrinsic property of tumors, and therefore, high-dose X-ray irradiation has to be used. Under these circumstances, radiotherapy faces the challenge of maximizing the therapeutic effect while minimizing adverse effects on surrounding healthy tissue. To address this issue, we have been focusing on the mechanisms of radioresistance in esophageal cancer while simultaneously looking for novel nanomaterials to enhance radiotherapy.^33^

Radiosensitizers can improve the selectivity of radiotherapy by differentiating the absorption of radiation between tumor tissue and healthy tissue. High *Z* materials, especially metal nanoparticles, act as sensitizing reagents for radiotherapy by increasing the deposition of X-rays at the tumor site. In 2018, Lin et al. first reported the application of Hf-based MOFs, i.e., Hf_12_-DBA and Hf_6_-DBA, as radiosensitizing agents; these agents were synthesized by solvothermal reactions.^19b^ Hf_12_-DBA, which has a sheet-like morphology, showed a good ablative effect on CT26 mouse tumors after 10 consecutive X-ray irradiations, but Hf_6_-DBA only slowed tumor growth under the same conditions.

To provide environmentally friendly Hf-based MOFs with good therapeutic effects, we synthesized UiO-66-NH_2_(Hf) nanoparticles with a size of 95 nm. In contrast to what has been reported previously, the synthesis of UiO-66-NH_2_(Hf) reported here was achieved by heating an aqueous solution of HfCl_4_ and the corresponding organic ligand. This synthetic process can easily be scaled up, as it does not involve organic solvents and high-pressure reactions, bridging the gap between laboratory studies and clinical applications.

Owing to the high content of the high-Z element Hf, UiO-66-NH_2_(Hf) has enhanced X-ray absorption with superior effects compared to the commercial CT contrast agent iodixanol, as reflected by *in vitro* and *in vivo* CT imaging results. Not unexpectedly, UiO-66-NH_2_(Hf) led to cell apoptosis by inducing cellular DNA damage and increased ROS levels, consistent with literature reports. In xenograft models, a single injection of UiO-66-NH_2_(Hf) in combination with a single fraction of X-ray irradiation significantly inhibited tumor growth. This single local treatment avoided the exposure of normal tissues to X-rays as much as possible and minimized the side effects of ionizing radiation.

In addition, due to the porosity of MOFs, the loading of functional components, such as drugs and photosensitizers, is easily achieved. We envision that the combination of radiotherapy sensitization with other therapies will further optimize the therapeutic effect, which will be investigated in the future.

## 5 Conclusions

In summary, we synthesized Hf-based MOF nanoparticles (UiO-66-NH_2_(Hf)) and investigated their role as radiosensitizers for radiotherapy for esophageal cancer. As a high *Z* element, Hf provides a large cross section for high-energy X-rays, thus increasing energy deposition in the tumor, which leads to DNA breaks and increased toxic ROS production in cancer cells, thereby resulting in apoptosis. The enhancing effect of UiO-66-NH_2_(Hf) on radiotherapy was well demonstrated in *in vitro* and *in vivo* experiments. Due to the features of inexpensive raw materials, simple and easy reactions, and no additional surface modifications, we believe that UiO-66-NH_2_(Hf) is an advanced nanomaterial closer to translation into clinical research than the numerous complex nanosystems previously reported.

## Supporting Information Available

Experimental materials, experimental instrumentations, scratch assay, and additional results (Figures S1–S5) (PDF)
