# Supplementary material for: Hafnium-Based Metal–Organic Framework Nanoparticles as a Radiosensitizer to Improve Radiotherapy Efficacy in Esophageal Cancer
Source: ACS Omega. 2022 Mar 30;7(14):12021–9. doi: 10.1021/acsomega.2c00223 (PMC9016869; doi:10.1021/acsomega.2c00223)
Supplement: Supplementary file 1 — ao2c00223_si_001.pdf [file ao2c00223_si_001.pdf]

Supporting information

Hafnium-Based Metal–Organic Framework Nanoparticles as a Radiosensitizer to Improve Radiotherapy Efficacy in Esophageal Cancer

Wei Zhou, Zhulong Liu, Nana Wang, Xue Chen, Xiaozheng Sun and Yufeng Cheng*

a. Department of Radiation Oncology, Cheeloo College of Medicine, Qilu Hospital, Shandong University, Jinan, China. *Email: qlcyf@sdu.edu.cn

Supplementary figures

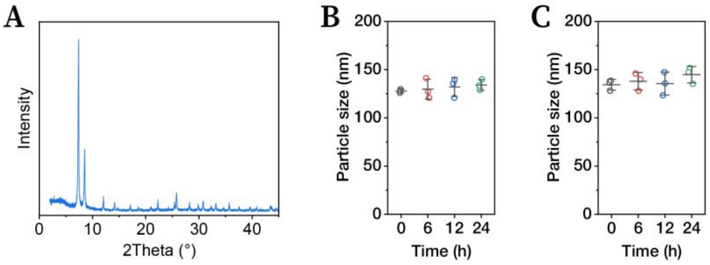

Figure S1. Stability of UiO-66-NH_2(Hf) nanoparticles. (A) PXRD pattern of UiO-66-NH_2(Hf) treated with PBS for 24 hours. (B) Time-dependent particle size in PBS based on DLS measurements. (C) Time-dependent particle size in DMEM based on DLS measurements.

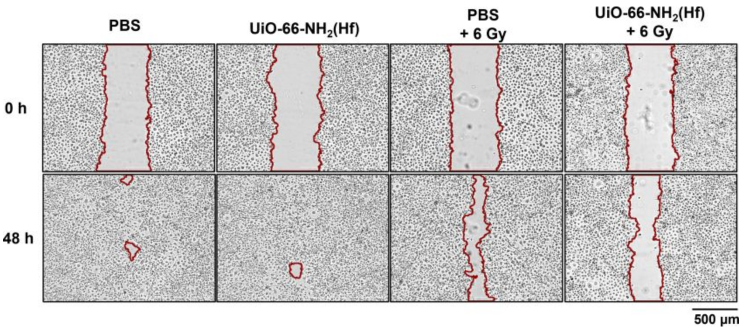

Figure S2. Scratch tests of KYSE 150 cells treated with UiO-66-NH_2(Hf) (0 or 50 µg/mL) for 4 h and exposed to 0 or 6 Gy of X-ray irradiation.

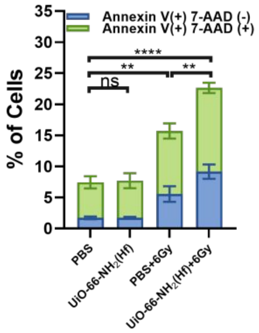

Figure S3. Proportion of annexin V positive cells in KYSE 150 cells incubated with UiO-66-NH_2(Hf) (0 or 50 μg/mL) for 4 h with or without 6 Gy X-ray irradiation. Data are presented as the mean ± SD, $n = 3$. Significance was determined by one-way ANOVA with Tukey's multiple comparisons test. **p<0.01; ****p<0.0001; *ns*, no significance (p>0.05).

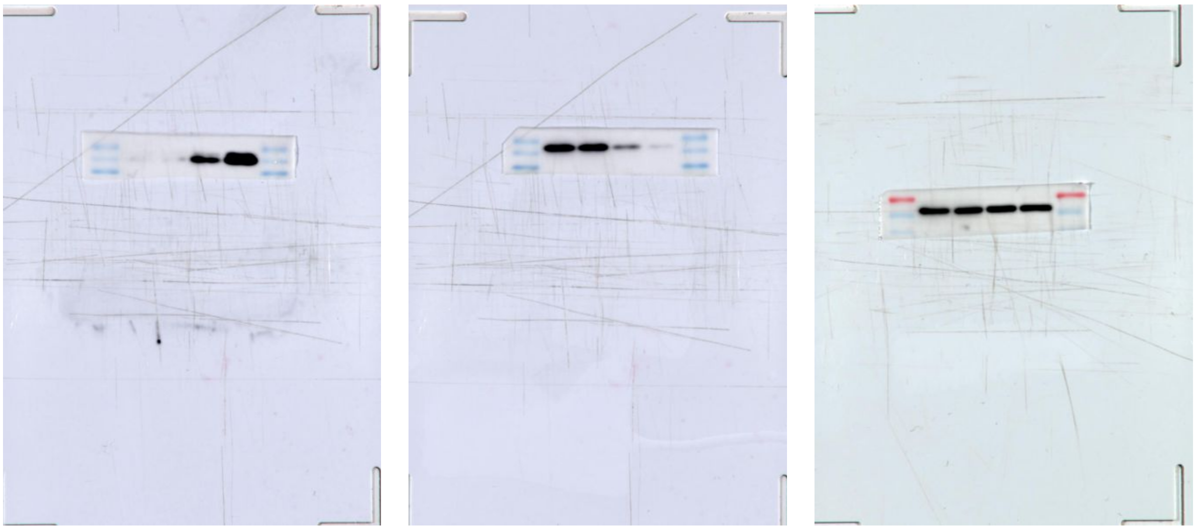

Figure S4. Uncropped western blotting images of Bax (left), Bcl-2 (middle), and tubulin (right).

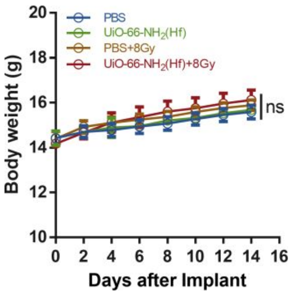

Figure S5. Body weight curves of KYSE 150 tumor-bearing nude mice. Data are presented as the mean ± SD, $n = 4$. Significance was determined by two-way ANOVA. *ns*, no significance (p>0.05).

Experimental materials

$HfCl_4$ was purchased from Macklin (Shanghai, China). 2-Aminoterephthalic acid was purchased from TCI (Shanghai, China). Ethanol and acetic acid were purchased from Sinopharm (Beijing, China). Dulbecco's modified Eagle medium (DMEM) and phosphate-buffered saline (PBS) was purchased from Gibco (Thermo Fisher Scientific Inc., USA). CCK-8 assay kit was purchased from Dojindo (Shanghai, China). Hoechst 33342 and 2',7'-dichlorodihydrofluorescein diacetate (DCFH-DA) was purchased from MedChemExpress (Shanghai, China). Annexin V-APC and 7-AAD apoptosis kit was purchased from Procell Life Science & Technology Co.,Ltd.

Experimental instrumentations

Transmission electron microscopy (TEM) images were recorded on a Hitachi HT7700 120 kV Compact-Digital Transmission Electron Microscope. Scanning electron microscopy (SEM) images were recorded using a Hitachi SU8010 instrument. Powder X-ray diffraction (PXRD) patterns were obtained on a Rigaku SmartLab SE X-Ray Powder Diffractometer with Cu Kα line focused radiation (λ = 1.5405 Å) from 2θ = 3.00° up to 50.00° with 0.01° increment. Inductively coupled plasma optical emission spectrometry (ICP-OES) measurements were carried out using a Thermo Scientific iCAP 7000 ICP-OES. Nitrogen-adsorption isotherms were measured at 77 K with a Micromeritics ASAP2020 HD88 Surface Area and Porosity Analyser. Before measurement, the samples were degassed in vacuum at 120 °C for 12 h. The Brunauer–Emmett–Teller (BET) equation was used to calculate the specific surface areas. Hydrodynamic particle size was measured using Malvern Zetasizer Nano ZS90 System. Laser scanning confocal fluorescence images were captured with a Leica TCS SP8 Confocal Laser Scanning Microscopy. Microplate assays were carried out on a Molecular Devices SpectraMax i3x Multi-Mode Microplate Detection System. Radiation therapy *in vitro* and *in vivo* performed on Varian Clinac 23EX linear accelerator.

Scratch assay

KYSE 150 cells were seeded into 6-well plates and grown to confluence. Cell monolayer was damaged by scratching with a sterile 200 μL pipet tip to obtain scratches. KYSE 150 cells were incubated with UiO-66-NH2(Hf) (0 or 50 μg/mL) for 4 h, and carefully washed with DPBS. The 0 h reference images of the scratched areas were taken using inverted microscope. Then, the cells were exposed to 0 or 6 Gy of X-ray irradiation. After additional 48 h incubation, the scratched areas were taken again. Cells treated with neither UiO-66-NH_2(Hf) nor X-ray irradiation were used as controls.
